# Supplementary material for: Trend and epidemiology of suicide attempts by self-poisoning among Egyptians
Source: PLoS One. 2022 Jun 16;17(6):e0270026. doi: 10.1371/journal.pone.0270026 (PMC9202942; doi:10.1371/journal.pone.0270026)
Supplement: Attachment — Submitted filename: Response-to-reviewers-comments.docx [file pone.0270026.s001.docx]

**Response to Reviewers’ Comments**

| **Reviewer’s comment** | **Response** |
| --- | --- |
| **Dear editor and reviewers, thanks so much for your great and endless effort. I hope to find the response satisfactory and admired** | |
| We note that your study included both retrospective and prospective elements. Please update the manuscript to include further details regarding the prospective element of your study. | Re-written and Explained line 95-102 |
| Please ensure you have specified the demographics and characteristics of the individuals you collected data from, the data collected as part of this study and how the results were used to inform the results described. Please also ensure you have clearly indicated how the data reported in Results section were collected. | Re-written and Explained line 103-147 |
| We also note that your study lists of the causes of suicide. Please ensure that you have specified how this was determined in your Methods section. | Re-written and Explained , line 134-137 |
| Thank you for including your ethics statement:  "Approval was obtained from local Institutional Review Boards (IRB) following the Declaration of Helsinki under ID."  Please amend your current ethics statement to include the full name of the ethics committee/institutional review board(s) that approved your specific study. | Re-written and added, line 149-150  The Research Ethical committee of Menoufia Faculty of Medicine, Menoufia University (ID: 2/2021COM) and the Research Ethical Committee of the Faculty of Medicine, Tanta University (ID: 34244/11/20). |
| The manuscript must describe a technically sound piece of scientific research with data that supports the conclusions. Experiments must have been conducted rigorously, with appropriate controls, replication, and sample sizes. The conclusions must be drawn appropriately based on the data presented. in addition to summary statistics, the data points behind means, medians and variance measures should be available. | Re-written and corrected |
| The title is not sufficiently focused more a short version of the abstract. | Changed  **Incidence, Distribution, and Determinants of Suicide by Self-poisoning in Two Egyptian Provinces** |
| The abstract is too long for an original paper and needs to be more structured based on the established format of the journal. | Get shortened line 20-43 |
| For an international readership it would be especially interesting to compare international trends to the situation in Egypt and the middle eastern region. I would suggest to take out general statements on suicide and refer to the literature and focus on describing the specific circumstances and risk factors for suicidal behaviour and suicide in the region. That should follow a clear structure in the introduction, so that the reader always knows what the authors are referring to. | Modified and changed line 50-87 |
| Critical factors like cultural aspects, religion, family structures and gender related expectations and risk factors are not described in a way, that allows the reader to be prepared for the results section. | Added line 79-82 |
| Also the fact, that suicidal behaviour and thoughts are highly stigmatized and the suicide rate might be under reported would be important for the conclusion and discussion later on. | Explained, line 283-287 |
| Also suicide rates in specific populations like street entrenched youth or rural populations are not reflected or presented. So the reader learns very little about specific vulnerable populations and risk factors. Also to study something is not a sufficient objective. I’m sure the authors had interests and ideas beyond that. | Explained, line 283-287  Line 316-321 |
| The method section again needs a clear structure like describing the study sample e.g how many patients in phase 1 and 2 etc., the evaluation methods, the timeframe and the expertise used and available to carry out the research and the detailed statistical procedures used. The governorates were not mentioned so the reader can not apply context knowledge. | Corrected line 90-146 |
| The terminology between self-poisoning and suicide is used in a confusing way. It should be clearly emphasized what the authors are talking about. | Corrected to suicide by self-poisoning |
| The lack of information and structure makes it complicated to understand the results. What constitutes the assessment of poisoning, Who is doing that and based on what criteria it is classified as suicide. | Re arranged line 168-217 |
| In case of the use of psychotropic substances often several substances are used at once but there is no mention of polysubstance use. That way the results section is extremely complicated to follow and unclear in its conclusions. A straight forward presentation of results should be well structured and allowing the reader to understand every step of the assessment! | Reordered in a more logic way |
| The discussion of the paper is including a lot of interesting and important considerations, which should already been mentioned in the introduction. Again it needs restructuring in order to be able to draw conclusions. | Added and corrected |
| The discussion is also not answering the question related to the increase in suicidal actions. Also there is a lack of comparison to the international development and the international literature. Is Egypt in a specific situation? Are there specific provinces which are worse off and why? Some comments are inconsistent: they mentioned suicide but their focus on self-poisoning  They mentioned that the study was along 4 years and, in the discussion, said 2019-2020 only. They stated in the results the total number of self-poisons was 3383 and in discussion only the suicidal number (2724). Needs clarification.  They did not name the governorates or give any background about them. They showed that the rates of their centers are much higher than the Egyptian national number without any rationalization.  The explanation of the third paragraph as a difference in the culture only needs to be more explained.  The explanation of the gender differences in the fourth paragraph as by hormonal causes only are not logic.  Same for the fifth paragraph, hormonal reason is reported as an explanation for suicide in students! In the eighth paragraph, I think these are risk factors for suicidal self-poisoning not causes of suicide. Risk factors have weak explanations. | Explained in detail line 222-320  The study was carried out over two phases :i. a retrospective phase including the data extracted from the medical records belonging to patients admitted to both centers and diagnosed as self-poisoning suicide during the years 2016, 2017, and 2018 (n=969, 1,192, and 2,258 respectively). These data were utilized to show the trends of suicide by self-poisoning and to generate the major themes mentioned as causes of suicide by self-poisoning during the salient period. ii. a prospective phase including all patients admitted to both centers and diagnosed as suffering from toxic exposure including accidental poisoning (n=659) and suicide by self-poisoning(n=2,724) conducted during the period from January 2019– to January 2020 (n=3,383).  The current study is a bi-designed study carried out during the period January 2016–January 2020 in two Egyptian poison control centers serving two Egyptian provinces, Menoufia and Gharbia governorates inhabited by more than 10,000,000 individuals. The main center at MPCC serves over 4,520,494 individuals over 2,543.03km2, and TPCC is the only center serving more than 5,145,595 individuals living over 1,942 km2  Explained in detail line 239-246  Explained line 297-305  Explained line 287-296 |
| Based on that the conclusions are not really conclusions because there are no recommendations or suggested consequences based on the results presented. I recommend the authors to collaborate with native speakers use more visuals in order to increase readability. | Corrected line 342-349 |
| Language has been edited by EKB | **Assignment number:** EKBEAW-1088  **Filename:** Manuscript for language editing_INQ-215870821_EKBEAW-1088.docx  **Primary Editor Name:** Delores |
