# Supplementary material for: Trend and epidemiology of suicide attempts by self-poisoning among Egyptians
Source: PLoS One. 2022 Jun 16;17(6):e0270026. doi: 10.1371/journal.pone.0270026 (PMC9202942; doi:10.1371/journal.pone.0270026)
Supplement: Attachment — Submitted filename: Response to Reviewers comments.docx [file pone.0270026.s002.docx]

**Response to reviewers’ comments**

The authors extend their appreciation for the reviewers for their great efforts in improving the quality of the manuscript. We found all proposed comments valuable and enriching the article. We hope we could fix the modifications meeting your expectations. Based on your valuable and comments, we changed the title and delineated the attempted from those died by suicide self-poisoning hoping to find satisfactory and clearly presented.

| **Reviewer comments** | **Response to reviewer comments** |
| --- | --- |
| **Reviewer 2** | |
| 1. Lines 24 and 25 mention, “the first part was conducted retrospectively to show the trend of suicide attempts over 2016, 2017, and 2018” - So, the 4419 patients mentioned here – are those who attempted suicide or those who died of suicide? I recommend that the data regarding patients diagnosed with suicide by self-poisoning and suicide attempt by self-poisoning is delineated and changed across the article (see point 8 below) accordingly. | The whole article was reframed to focus on the suicidal attempt as per your valuable suggestion.  This modification is reflected in the methods, results and discussion in different locations. |
| 2. Line 60 – “Reports published in the US considered suicide by self-poisoning the most common method of suicide attempts among adolescents (66.5%)”. I recommend removing suicide by self-poisoning and replacing it with self-poisoning as the article refers to suicide attempts and not suicide. | Fixed as mentioned. In the introduction section:  “The reports issued from the United States considered the self-poisoning as the most common way of attempting suicide among youths [9]” |
| 3. Line 77 – The sentence does not clearly communicate the author’s thoughts to the reader. Can you clarify “drugs?” I recommend rewriting the sentence for clarity. | Fixed as mentioned. In the introduction section:  “The residence and age of patients attempting suicide influence the type of the material they used to attempt suicide. Though youth victims prefer the xenobiotics, pesticides are commonly used by older subjects living in rural areas [14]” |
| 4. Line 83-87 – The sentence is too long and loses clarity. I recommend breaking it into two sentences. | Fixed as mentioned. In the last section of the introduction:  Previous studies investigating suicide attempt by self-poisoning in Egypt are grossly inadequate and are built on previous figures which may be misleading or inaccurate [15]. This work aimed to assess the trend, incidence, and risk factors of suicide attempts by self-poisoning among Egyptians by examining the cases admitted to two main poison control centers. |
| 5. Line 97 – Same question as point 1. Does 969, 1,192, and 2,258 refer to patients who died of suicide or are these the number of patients who attempted suicide. Based on this the diagnosis should reflect suicide by self-poisoning versus suicide attempt by self-poisoning | Fixed. Number of cases is clearly presented in the flow chart (Fig 1). Analysis of the trend included all cases of suicide attempt by self-poisoning (excluding fatalities). Also, analysis of the last year (January 2019-January 2020) was carried out among suicide attempters only. However, to be more relevant, we used deaths only to calculate the rates as mentioned at table 1. |
| 6. Line 101- Why was accidental poisoning included  ? Is there a relevance to suicide which is the primary objective of the study? | Patients with accidental poisoning were recruited to provide a benchmark by comparing the incidence and associated risk factors with attempted suicides by self-poisoning. However, they were not counted in trend analysis. Flow chart clearly identifies this. |
| 7. The lines 129-146 under data collection tools are confusing to the reader. Do these lines refer to patients who attempted suicide by overdose? Clarifying point 1 above and rewriting this section is recommended. | This section (data collection tools) was completely rephrased under the methodology section. I hope it is more clarified now. |
| 8. Lines 169-171, 173, 175, 186 and table 1, 222, 247, 274, 288, 298, 307, 316 and 343 – does this data pertain to suicide attempts or to suicide? If it refers to suicide attempts, then the sentences need to be changed to reflect that. | Clarified in all relevant sections in the whole manuscript |
| 9. Line 224 – the article refers to suicide attempts and not suicide as mentioned in this line. Recommend changing it for accuracy. | Fixed and replaced with relevant reference (Spirito A, Esposito-Smythers C. Attempted and completed suicide in adolescence. Annu Rev Clin Psychol. 2006;2: 237–266.) as follows:  “…..These numbers are much higher than that reported in 2004, when 2.9% of adolescents attempted suicide that need medical emergency. However, part of this great gap is attributed to the different sampling between different studies. Our study targets those admitted to poison centers while the other study calculated this rate considering the general population [20]” |
| 10. Line starting 337 ending in 339 is incomplete and I recommend rewriting it to convey accurate meaning. | Rephrased as per request under the strength and limiattaion section as follows:  “Studying many issues such as the follow-up of survivors to check if cases are referred for psychological counseling, the effects on families, and if there are any other associated or hidden factors to avoid repetition of the suicide event was out of our hands due to the sensitivity of this subject and associated stigma within the families of the victims” |
| **Reviewer 3** | |
| - Not always easily readability of the text and makes it difficult to follow at times. | We have sent the manuscript for a native language proofreader to rephrase the difficult parts. I hope it is more clear and easily readable now. |
| - In line 47, it is not clear if the authors are implying suicide being the leading cause of death in children and adolescents or suicide deaths by numbers in this age group. | Suicide being the leading cause of death in children and adolescents. It was clarified in the sentence after as follows:  “The suicide worldwide 2014 report by WHO states that suicide is the second leading cause of death among 15-29 year [1].” |
| - In line 48, the authors report a steady increase in suicide rates globally (WHO 2001 data). However, new data in the Suicide worldwide 2019 report by WHO (published in 2021) states overall the global age-standardized suicide rate is somewhat in decline (except some regions, for example, the Americas). Ref: https://www.who.int/publications/i/item/9789240026643 | We add the proposed reference and rephased the sentence to be as follows:  “Although the Suicide worldwide 2019 report by WHO states overall decline in the global age-standardized suicide rate (except some regions, for example, the Americas), the global age-standardized suicide rates varied between countries from fewer than two deaths by suicide per 100 000 to over 80 per 100 000 [2]. Most deaths by suicide occurred in low-and-middle-income countries [3].” |
| - Authors rightly report stigma surrounding the suicide. In line 303, the authors suggest gender differences in suicide rates to hormonal or interpersonal conflict. The full comprehension of the gender paradox in suicidal behavior requires further research. Suggestion for authors to review studies on gender differences in suicide and other possible risk factors including but not limited to psychiatric disorders.  Ref: Qin, P., Agerbo, E., Westergård-Nielsen, N., Eriksson, T., & Mortensen, P. (2000). Gender differences in risk factors for suicide in Denmark. British Journal of Psychiatry, 177(6), 546-550. doi:10.1192/bjp.177.6.546.  Tóth MD, Ádám S, Birkás E, Székely A, Stauder A, Purebl G. Gender differences in deliberate self-poisoning in Hungary: analyzing the effect of precipitating factors and their relation to depression. Crisis. 2014;35(3):145-53. doi: 10.1027/0227-5910/a000245. PMID: 24491825. | Right. We elaborated on this aspect and added the mentioned references, besides another recent reference form Egypt. This part in the discussion was corrected as follows:  Right. We elaborated on this aspect and added the mentioned references, besides another recent reference form Egypt. This part in the discussion was corrected as follows:  “The current study depicted that female were at a significantly higher risk than male regarding attempting suicide by self-poisoning. A similar picture is seen globally, where self-harm ranks the second among females and the third among males[37,46]. A recent study carried out in Egypt conveyed that two thirds of suicidal self-poisoning happened among females [47]. In an Australia, females attempting suicide by self-poisoning outnumbered males (3:1) [22]. Females tend to consume already available materials at home, while males tend to swallow a substance kept outside or purchase it[48]. The availability of the substance, which is mainly pesticide, in addition to hormonal disturbances and the usual tendency of females to conflict with their guardians are the main causes for attempting suicide by self-poisoning [49–51].The restricted customs in developing countries, and the burden of spinsterish on females, besides the low socioeconomic standards and low wages predispose for the high prevalence of suicide attempt by self-poisoning in females [47].  Qin et al elaborated on the suicide attempt gender variations. Following psychiatric disorders, living in urban areas, with no children, and the presence of family history of suicide raise the risk of suicide in females compared to males [52]. Tóth et al., examined the gender differences in suicide attempts. There was gender in depression presentation among patients with interpersonal conflicts. Females with interpersonal conflicts who attempted suicide by self-poisoning showed higher levels of depression than males [53].” |
| - In line 293, the authors report risk factors in less than 25 years old population. It is unclear if authors are implying social media as the monster or a specific online site named the monster. | The word (monster) was deleted for confusion |
| - Lines 296, 300, 303 have missing references. | The corresponding references for the mentioned sections were added as per your request  Egypt is in the advanced rank of internet usage in middle east and exhibits high smart phone usage among young people (90%) [45].  In an Australia, females attempting suicide by self-poisoning outnumbered males (3:1) [22].  Females tend to consume already available materials at home, while males tend to swallow a substance kept outside or purchase it[48]. |

**A**
