# Supplementary material for: Trend and epidemiology of suicide attempts by self-poisoning among Egyptians
Source: PLoS One. 2022 Jun 16;17(6):e0270026. doi: 10.1371/journal.pone.0270026 (PMC9202942; doi:10.1371/journal.pone.0270026)
Supplement: Attachment — Submitted filename: Response to Revierwers comments.docx [file pone.0270026.s003.docx]

**Response to reviewers’ comments**

**Thanks so much for your valuable comments that enriched the article. We responded to all comments and we hope you find it satisfactory and admired.**

**Reviewer #3:**

The authors have adequately addressed comments raised in a previous round of review and feel that this manuscript is now acceptable for publication except for multiple grammatical errors. I encourage authors to fix these before publication.

Suggestion for authors: can use online freely available software like Grammarly, etc.

We have sent the manuscript to a proofreader who had checked for any errors or mistake. However, we didn’t highlight the language changes as they were many, to avoid confusing the other responses to reviewer comments.

**Reviewer #4:**

1. Abstract: authors present results from the univariate logistic regression models. However, the multivariate model is more appropriate for final results, since this accounts for other variables included in the model.

Corrected as per request.

2. In the subjects and sampling section of the Methods, authors state that fatalities were excluded from the analyses of risk factors. Authors should provide additional justification for this decision.

Totally agree with this point. Unfortunately, exclusion of fatalities from analysis was based on the reviewer requirement in the last round of revision. However, we had deleted the statement” fatalities were excluded from the analyses of risk factors”, and we included the fatalities in the analysis as revealed at Table (2). Besides, we elaborated in this part in the abstract, results, discussion, especially the point referring for significantly higher fatalities due to self-poisoning by Aluminum phosphide.

3. Some of the information collected on the attempts are likely known about the fatalities. At a minimum, authors should provide some information about how the characteristics (age, gender, other variables known) of those who died by suicide in 2019 compared to those who attempted suicide that year but were unsuccessful.

The authors, as per the request, conducted the test of significance to compare characteristics (age, gender, other variables known) of those who died by suicide in 2019 compared to those who attempted suicide that year. A table was added (Table 2).

4. In the inclusion criteria section, authors state that all patients admitted to poison control centers with complete medical records were eligible for the study. Authors should provide information about poison control centers in Egypt so that readers understand who the population under study is. For example, are all cases of suspected poisoning referred to poison control centers (including deaths)?

Agree. This part was rephrased completely in the section of the methodology under the subheading Inclusion criteria. The route of admission to poison control centers and triaging the patients were mentioned in more comprehensive way. We hope it meets your expectations.

5. In the Statistical Analysis section, authors should describe their approach for model building - how did they go from the univariate model to the multivariate model? Was overlap/collinearity between variables considered?

This part was fixed and rephased with a reference. Based on Bursac et al., 2008, the best way to select the included varaibles is to subject all significant variables in the univariate logistic regression to multivariate logistic regression to detect the riskiest factors to attempted suicide by self-poisoning.

6. Univariate analysis section of results: Authors do not mention the finding for medium SES.

Detailed results of univariate logistic regression, including the medium SES was reported in the results section as per request.

7. Multivariate analysis section of results: why do the authors combine categories for variables in the multivariate models? This needs to be justified and explained.

Based on that comment, and to allow simple and easy interpretation, we do uncombined all variables and re-performed logistic regression as revealed at Table (4) and the corresponding part in the results section.

8. Authors spend several paragraphs in the Discussion about females attempting suicide, yet females are not significantly more likely than males to attempt suicide in the multivariable model.

Right. The multivariate model didn’t outline being a female as one of the significant suicide attempt risk factors, as this model highlight the most significant predictor considering the other variables which showed the riskiest factors like SES, age , residency and occupation. However, we could not overlook the significance of gender seen in the univariate analysis and when comparing studied patients according to the successfulness and manner of exposure (attempted versus successful, and accidental versus suicide self-poisoning). 62% of the Self-poisoning attempters, and 79.6 were females (which constituted more than half of studied Patients. Moreover, the added paragraphs were response to the reviewer comments in the first and second rounds of revisions.

the
Minor points:

1. Abstract: "The death rate due suicide" should be "The death rate due to suicide" and "which raise a serious concern" should be "which raises a serious concern". Also, to be consistent with later presented results in the manuscript, "CI95%" should be "95% CI"

The Confidence interval 95%
was fixed at the different sections of the text aligned with the results, as per request.
2. Introduction: "among 15-29 year" should be "among people aged 15-29 years" and "2nd cause of death" should be "2nd leading cause of death"

Fixed

3. Inclusion criteria: "For example, cut the wrists" is an incomplete sentence.

Fixed

4. Data collection tools: "which was use in trend analysis" should be "which was used in trend analysis", "Confirmed by clinical evaluation..." is an incomplete sentence, and "age, gender, etc." should list all variables collected, not just saying "etc."

Fixed

5. Statistical Analysis: "Qualitative data was" should be "Qualitative data were" and "Calculated by dividing..." is an incomplete sentence.

Fixed

6. Figures 3 and 4: in the legends "slef-poisoning" should be "self-poisoning" and "self-poisoning" should be "self-poisoning". Also, authors should clarify what is meant by total cases - is this attempted + successful?

Fixed. The total number of suicides by self-poisoning reflects the sum of attempted and successful suicide by self-poisoning. This clarification was added to the mentioned figures’ ligands.
7. Results, 1st paragraph: "attempted suicide by suicide" should be "attempted suicide by self-poisoning"

Fixed

8. Table 1: the denominator written in the table for proportionate mortality rate does not seem correct, as this is the same as what is written for the self-poisoning suicide death rate.

Fixed, it was typing error.

9. Table 2 (and related text in Results): The percentages in the text of the paragraph at the bottom of page 7 (for aluminum phosphide and drugs used in attempted suicide) do not match what is presented in Table 2. Authors should also clarify in the text and the table that the percentages for the drugs are calculated out of the 467 individuals who used drugs.

Sorry for this mistake due to reanalysis of data based on reviewers’ comments, there was unintended mismatching between table and text which was figured out after submission. However, the numbers and percentages of Causative Agent used as mentioned at Table (2), is calculated out of 2724 as highlighted in the title of the table, and not out of 467. We used the word causative agent to include the different categories of used substance in terms of chemicals, drugs, pesticides,….etc. However, a brief hint was added to the text clarifying the overall number of cases included in the calculation.

10. bottom paragraph of page 7: "was pesticides (38.7%) then aluminum..." should be "was pesticides (38.7%), then aluminum..."

Fixed

11. Univariate analysis results: the OR written for age<25 years does not match Table 3. Based on the numbers presented, it seems as though the number in the text is probably correct, while there is a typo in Table 3. Authors should carefully check the numbers and make the correction wherever it is needed. "female student" should just be "student" and the OR and CI for not working should be corrected (since the authors have re-written the OR and CI for student). "patients Those" should be "individuals" or "patients" and "confidence interval" should be "95% CI" to be consistent with other presented results in this section.

The entire section was revised and corrected as per your suggestion.

12. Discussion, 1st paragraph: "The rate of suicide attempts in the general..." should be "The rate of suicide attempts by self-poisoning in the general..." In this same paragraph, authors compare the result to a study of teens and make a point that the rate is much higher in 2019 than 2004. It is unclear why this comparison is relevant if the 2004 study is just about teens, while the current study is about all attempts.

This paragraph was rephrased and fixed to alleviate the confusion. We fixed the mentioned words. Regrading specifying the teens, this was requested by the reviewer in the second round of revision, considering the significance of suicide by self-poisoning among the patients aged less than 25 in the current study. Moreover, we had added a reference supporting the findings of suicide by self-poisoning among teens. Then, in the next paragraph, we discussed the problem of suicides by self-poisoning among admissions without reflecting on specific age group. We hope that meets your expectations.

13. Discussion, 2nd paragraph: "Suicides by self-poisoning reached 28.76" should be clarified. First, authors should state whether this is out of admissions or population (/1000 or /100000). Second, authors need to clarify if this is attempts, deaths, or attempts+deaths. Fixed

14. Last sentence on page 12 ("Examining several issues...") is an incomplete sentence. Fixed

15. Conclusion: authors do not mention financial reasons, which was more common than psychological reasons for the attempted suicide. Also, the summary sentence on the results from the logistic model needs to be rewritten, since the authors have combined variables together (students under the age of 25, for example), while the model looks at each variable while holding the other variables constant (so authors can say, "students, those under the age of 25 and those who live in a rural area..."

Fixed
