# Supplementary material for: Trend and epidemiology of suicide attempts by self-poisoning among Egyptians
Source: PLoS One. 2022 Jun 16;17(6):e0270026. doi: 10.1371/journal.pone.0270026 (PMC9202942; doi:10.1371/journal.pone.0270026)
Supplement: Attachment — Submitted filename: Response to Revierwers comments.docx [file pone.0270026.s004.docx]

**Response to reviewers’ comments**

**Reviewer #3 and Reviewer #4:**

Thanks so much for your support and great efforts to make our article presented in the best way.

Reviewer 4#: The authors have addressed the majority of my earlier concerns. There just remain a few very minor points that should be corrected:

1. on page 8, 1st full paragraph beginning with "Out of the 2724 cases...": authors refer to female students less than 25 years old are at higher risk of death from suicide when referring to results in Table 2. The results would suggest that females have increased odds of death relative to males, students have higher odds of death than those working, and those <=25 have higher odds of death than those >25. So interpreting the results as the combination of those variables (female students less than 25) is not exactly consistent with the analysis. Authors should rephrase this to indicate that sex, occupation, and age are separate variables.

Response: Thanks so much and we apologize for this misinterpretation. We corrected it as per your request on individual basis. Fortunately, this part was interpreted on individual basis on the discussion section as previously recommended.

2. Also on page 8 in the Univariate analysis section, authors said in their response that they corrected the repeated OR and CI, but that is not reflected in the submitted clean or corrected version with changes highlighted. The OR for not working is written exactly the same as the OR for student, but Table 3 shows these are in fact different ORs and CIs. Authors should put the correct OR and CI for "not working" in the text.

Response: Thanks so much and we apologize for this mistake (typo), and we corrected it. OR and CI were written separately for both students and not working

3. In the 1st paragraph of the Discussion, authors added a percentage (37.4%) for the 1196 attempts made by individuals less than 25 - authors should recheck that percentage, since 1196/2523=47.4 and 1196/ (2523+201) =43.9, so it is not clear where the 37.4% is coming from.

Response: Thanks so much and we apologize for this typing error, and we corrected it from 37.4 to 47.4%
4. Table 1: authors should include a note about where the 1424 (corrected denominator for the proportionate mortality rate) comes from or what it refers to

Response: Right. This part was fixed in the results section, and as a foot note under Table (1).

The equation of proportionate mortality rate = number of deaths (suicide) due to self-poisoning divided by deaths from all causes. The total number of deaths during this year from all causes in the referred hospitals where the poising centers present equaled 1424.

Proportionate mortality rate: Deaths caused by a particular cause/Deaths from all causes
